# Cerebrospinal Fluid Phosphorylated Alpha‐Synuclein in Newly Diagnosed Parkinson's Disease

**DOI:** 10.1111/ene.70167

**Published:** 2025-06-02

**Authors:** Camilla Christina Pedersen, Guido Alves, Ole‐Bjørn Tysnes, Jodi Maple‐Grødem, Johannes Lange

**Affiliations:** ^1^ Centre for Movement Disorders Stavanger University Hospital Stavanger Norway; ^2^ Department of Chemistry, Bioscience and Environmental Engineering University of Stavanger Stavanger Norway; ^3^ Department of Neurology Stavanger University Hospital Stavanger Norway; ^4^ Department of Clinical Medicine University of Bergen Bergen Norway; ^5^ Department of Neurology Haukeland University Hospital Bergen Norway

**Keywords:** alpha‐synuclein, biomarker, Parkinson disease, phosphorylated, SIMOA

## Abstract

**Background:**

Alpha‐synuclein (α‐syn), phosphorylated at serine 129 (pS129‐α‐syn), is a potential biomarker for Parkinson's disease (PD) because it is the predominant α‐syn species found in Lewy bodies.

**Methods:**

We developed an in‐house SIMOA assay, using commercially available components, to quantify pS129‐α‐syn in CSF. The clinical utility of the assay was tested in CSF from 120 patients with PD from the Norwegian ParkWest longitudinal study and 29 normal controls. Prior measurements of CSF total (t)‐α‐syn and the pS129‐α‐syn/t‐α‐syn ratio were included for comparison.

**Results:**

The lower limit of quantification of the in‐house assay used to analyze CSF samples from participants was 0.57 pg/mL, which was well below the observed concentrations of endogenous pS129‐α‐syn in CSF. Median CSF pS129‐α‐syn levels were slightly lower in PD patients compared to controls (5.7 pg/mL vs. 6.5 pg/mL), but the difference was not significant in the unadjusted (*p* = 0.404) or adjusted analyses (*p* = 0.270). There was no difference in the pS129‐α‐syn/t‐α‐syn ratio between patients and controls. Using linear mixed‐effects models, we found no association between baseline pS129‐α‐syn or the pS129‐α‐syn/t‐α‐syn ratio and motor or cognitive decline over a 10‐year period.

**Conclusion:**

We developed and applied an in‐house SIMOA that reliably quantifies pS129‐α‐syn in CSF samples. This study does not indicate a role for CSF pS129‐α‐syn or the pS129‐α‐syn/t‐α‐syn ratio as clinically useful diagnostic or prognostic biomarkers in PD.

## 1 Introduction

Alpha‐synuclein (α‐syn) is a small neuronal protein that is predominantly located in the presynaptic region and plays a role in synaptic vesicle trafficking [1]. In Parkinson's disease (PD), α‐syn abnormally aggregates into inclusions called Lewy bodies (LB) and Lewy neurites that are a pathological hallmark of PD [2, 3].

A major posttranslational modification (PTM) of α‐syn is phosphorylation at serine 129 (S129) [4]. Phosphorylation of α‐syn at S129 (pS129‐α‐syn) is the predominant form of α‐syn found in LB, with over 90% of monomeric α‐syn found in LB being phosphorylated at S129 compared to less than 5% under physiological conditions [5].

Only a few studies have quantified pS129‐α‐syn in cerebrospinal fluid (CSF) samples from patients with PD and controls, with varying results [6]. While some studies have found increased levels of pS129‐α‐syn [7, 8, 9] in CSF from patients with PD compared to controls, others have found no differences [10, 11, 12, 13, 14, 15]. Notably, almost every study employed in‐house developed assays for pS129‐α‐syn, as commercial alternatives are scarce.

PS129‐α‐syn is a low‐abundant protein in CSF. SIMOA technology offers superior sensitivity and reproducibility compared to conventional enzyme‐linked immunosorbent assay (ELISA) methods for protein biomarker quantification [16]. One recent study, also utilizing the ultrasensitive single‐molecule array (SIMOA) technology to quantify pS129‐α‐syn, found lower levels in patients with PD [17], in contrast to previous findings [7, 8, 9, 10, 11, 12, 13].

To assess pS129‐α‐syn in CSF in our cohort of newly diagnosed patients with PD, we developed and validated a SIMOA assay using only commercially available components, which facilitates interlaboratory reproducibility. We used this assay, along with previously published data of CSF t‐α‐syn [18], to evaluate the diagnostic and prognostic ability of pS129‐α‐syn and the pS129‐α‐syn/t‐α‐syn ratio in a population‐based cohort of newly diagnosed PD patients with long follow‐up periods.

## 2 Materials and Methods

### 2.1 Patients and Controls

One hundred and twenty newly diagnosed patients with PD were included from the population‐based, longitudinal, multicenter ParkWest study [19]. The United Kingdom Brain Bank criteria [20] was used to diagnose all patients, who were also assessed using a uniform program including the Unified Parkinson's Disease Rating Scale (UPDRS) Part III [21], Hoehn and Yahr (H&Y) staging [22], and the Mini‐Mental State Examination (MMSE) [23]. Follow‐up UPDRS III scores were included for annual visits. Follow‐up MMSE scores were included for Years 1, 3, 5, 7, and 9. The Norwegian Regional Committee for Medical and Health Research Ethics in Western Norway approved the ParkWest study.

Twenty‐nine normal controls, none of whom had suspected neurodegenerative disease, were also included in this study. This group consisted of people who underwent either elective neurologic examinations or orthopedic surgeries at Stavanger University Hospital, one of the sites for the ParkWest study. Basic demographic data and MMSE scores were obtained. Procedures to collect clinical data and biological samples from the control subjects were also approved by the Norwegian Regional Committee for Medical and Health Research Ethics in Western Norway.

All participants signed written informed consent.

### 2.2 CSF Samples and pS129‐α‐Syn Measurements

CSF samples were obtained using standardized lumbar puncture procedures [24]. Samples were then centrifuged and stored at −80°C in polypropylene tubes. All samples underwent two freeze–thaw cycles for aliquotation purposes. We developed an in‐house SIMOA assay to detect CSF pS129‐α‐syn using the reagent preparations, optimizations, and validation steps described in the Supporting Methods and Results.

The lower limit of quantification (LLOQ) of the assay used to analyze CSF samples from patients with PD and controls was 0.57 pg/mL. The inter‐ and intraplate coefficients of variability (CV), assessed using quality control CSF samples present on each plate, were 15% and 9.5%, respectively. Sample concentrations ranged from 2.2 to 16.9 pg/mL. Sample CV ranged from 0.05% to 45.1%. Less than 9% of all samples had a CV% above 20%, and only three samples exceeded 30%.

### 2.3 Assay Procedure

Calibrators were prepared by four‐fold serial dilution of recombinant pS129‐α‐syn (RP‐004; Proteos, Kalamazoo, MI, USA) in Sample Diluent A (#101575; Quanterix, Billerica, MA, USA), ranging from 0.6 to 2,500 pg/mL. The 2,500 pg/mL calibrator was effectively above the detection limit of the SR‐X instrument and omitted from analysis. CSF samples were diluted two‐fold in the same diluent. One hundred microliters of calibrators and diluted CSF samples were added to the wells of a 96‐well plate (#101457; Quanterix). 25 μL 0.02 × 10^9^ beads/mL coated with anti‐phospho‐α‐synuclein Rabbit monoclonal antibody (#87281; Cell Signaling Technology, Danvers, MA, USA) were added to the wells, and the plate was incubated at 30°C for 30 min with shaking (800 rpm).

Plates were washed using the three‐step assay wash program on the microplate washer. Then, 100 μL of 0.3 μg/mL biotinylated anti‐alpha‐synuclein Mouse monoclonal antibody (#Ab280382; Abcam, Cambridge, UK) in detector diluent (#101359; Quanterix) was added to the wells before a 10‐min incubation at 30°C with shaking (800 rpm). After washing, 100 μL of 150 pM SBG concentrate (#103397; Quanterix) in SBG diluent (#100376; Quanterix) was added to the wells. The plate was then incubated at 30°C for 10 min with shaking (800 rpm). After the washing program was complete, plates were left to dry for 10 min before they were transferred to the SR‐X instrument (#102917; Quanterix). A vial of resorufin β‐galactopyranoside (RGP; #101736, Quanterix) was incubated at 30°C for at least 1 h with shaking (800 rpm) before loading the SR‐X. Curve fit and concentration calculations were performed in the SR‐X software version 1.2.0 (Quanterix), and results were exported to Excel.

Two quality control samples were included on each plate. These quality control samples were anonymized CSF obtained from people who underwent elective lumbar puncture as part of their clinical work‐up.

### 2.4 Inclusion of Previously Published CSF Measurements

T‐α‐syn levels of a subset of PD patients and controls were published in 2019 [18] and used in this study to calculate the pS129‐α‐syn/t‐α‐syn ratio. Three controls from this t‐α‐syn dataset had no available samples for pS129‐α‐syn analysis.

### 2.5 Statistical Analysis

STATA 18.0 was used for the analysis of demographic and clinical data. Normal distribution of continuous variables was assessed using the Shapiro–Wilk test. Normally distributed continuous variables were reported as mean and standard deviation (SD), and between‐group differences were evaluated using the Student's t‐test. All other continuous variables were reported as the median, along with the 25th and 75th percentiles, and between‐group differences were evaluated using the Mann–Whitney U test. Categorical variables were reported as frequencies and percentages, and differences were assessed using the chi‐squared test. CSF biomarkers, pS129‐α‐syn and t‐α‐syn, were transformed using the natural log to achieve a normal distribution for further analyses. The pS129‐α‐syn/t‐α‐syn ratio is included in further analyses as percentages.

Robust linear regression was used to evaluate the associations between pS129‐α‐syn and either diagnosis of PD or clinical variables, with adjustment for age and sex. Data for t‐α‐syn levels and the pS129‐α‐syn/t‐α‐syn ratios were only available for a subset of patients (*N* = 75), all without CSF blood contamination, defined as hemoglobin levels > 200 ng/mL [25]. For pS129‐α‐syn, analyses were repeated after excluding patients with CSF blood contamination (*N* = 29) or for whom data regarding blood contamination were not available (*N* = 6). *R* ^2^ values for all robust regression analyses were calculated using the rregfit package in STATA.

Linear mixed‐effects models were used to evaluate the effect of baseline pS129‐α‐syn and the pS129‐α‐syn/t‐α‐syn ratio on motor and cognitive decline, measured by UPDRS III and MMSE, respectively. All models were adjusted for age at baseline, sex, and time from baseline in years. Patient identifiers (intercepts) and time points (slopes) were included as random effects in all models, and first‐order autoregressive residual covariance structures were applied. For the pS129‐α‐syn/t‐α‐syn ratio model, the number of patients was limited to only those with confirmed hemoglobin levels below 200 ng/mL. The transformation described by Philipps et al. was applied to MMSE in order to reduce the ceiling/floor effect and curvilinearity of the raw scores [26]. For models including UPDRS III as the outcome, LME analysis was repeated to adjust for measures of total levodopa equivalent dose (LED) over 10 years. Models including MMSE score were adjusted for years of education at baseline. Reported marginal predictions for all models were calculated using the STATA margins command, and predicted plots were generated using the margins plot command. Nakagawa's marginal and conditional pseudo‐*R* ^2^ values for all linear mixed‐effects models were calculated using the r2_nakagawa package in STATA [27].

For all analyses, two‐tailed *p* values < 0.05 were considered to be significant. Adjustment for multiple testing was not applied.

## 3 Results

### 3.1 Baseline Demographic and Clinical Characteristics

In this study, we included 120 patients with PD and 29 normal controls. Baseline demographic and clinical characteristics and pS129‐α‐syn levels are presented in Table 1. There were no significant differences in sex, age, years of education, or MMSE score between patients and controls.

**TABLE 1:** Baseline demographic and clinical characteristics of patients and controls included in this study.

Clinical variables^a^	PD	Controls	*p*
*N* Total	120	29	
Male, *N* (%)	78 (65.0)	15 (51.7)	0.185
Age, years, Mean (SD)	67.1 (9.4)	67.8 (8.4)	0.750
Education, years	11.0 (9.0–13.0)	*N* = 27 10.0 (8.0–13.0)	0.201
MMSE score	28.5 (27.0–29.0)	29.0 (28.0–29.0)	0.112
Time since diagnosis, days	37.5 (20.5–57.5)	—	—
Time since first PD motor symptoms, years	1.6 (1.2–2.7)	—	—
UPDRS III	20.0 (15.0–29.0)	—	—
H&Y, Mean (SD)	1.86 (0.6)	—	—
CSF pS129‐α‐syn, pg/mL	5.8 (4.3–7.4)	6.5 (4.2–9.3)	0.404
CSF pS129‐α‐syn, pg/mL Hemoglobin < 200 ng/mL	*N* = 85 5.6 (4.3–7.2)	6.5 (4.2–9.3)	0.293
CSF t‐α‐syn, pg/mL^b^	*N* = 75 416.4 (290.1–508.9)	493.7 (361.5–618.0)	**0.039**
CSF pS129‐α‐syn/t‐α‐syn, %^b^	*N* = 75 1.4 (1.2–1.7)	1.4 (1.1–1.7)	0.905

*Note:* Bold *p* values are statistically significant at *p* < 0.05.

Abbreviations: BL, baseline; H&Y, Hoehn and Yahr stage; MMSE, mini‐mental state examination; N, number of participants; PD, Parkinson's disease; pS129‐α‐syn, phosphorylated alpha‐synuclein at serine 129; SD, standard deviation; t‐α‐syn, total alpha‐synuclein; UPDRS III, Unified Parkinson's Disease Rating Scale Part III.

^a^ Reported as median (25th–75th percentile) unless otherwise stated.

^b^ CSF total‐α‐syn was published earlier (Førland, Tysnes et al. 2020). All samples with confirmed hemoglobin levels < 200 ng/mL.

Robust linear regression was used to evaluate the associations between pS129‐α‐syn and clinical variables. PS129‐α‐syn had an association with age and t‐α‐syn at baseline, in both patients and controls (Table 2). Hemoglobin contamination is a concern for CSF t‐α‐syn analysis; after the exclusion of samples with hemoglobin levels above 200 ng/mL, the above associations remained significant.

**TABLE 2:** Baseline associations between log‐transformed pS129‐α‐syn and variables in patients and controls included in this study.

Variables^a^	PD			Controls		
	*β* (95% CI)	*p*	*R* ^2^	*β* (95% CI)	*p*	*R* ^2^
Sex	0.02 (−0.12 to 0.16)	0.775	0.12	−0.03 (−0.44 to 0.39)	0.892	0.16
Age	0.02 (0.01 to 0.02)	**< 0.001**	0.12	0.03 (0.00 to 0.05)	**0.047**	0.16
MMSE score	−0.01 (−0.05 to 0.02)	0.371	0.13	−0.18 (−0.40 to 0.04)	0.097	0.26
UPDRS III	−0.00 (−0.01 to 0.01)	0.529	0.12	—	—	
H&Y	−0.09 (−0.21 to 0.03)	0.136	0.14	—	—	
t‐α‐syn	0.45 (0.29 to 0.60)	**< 0.001**	0.35	0.69 (0. 26 to 1.12)	**0.003**	0.40

*Note:* Bold *p* values are statistically significant at *p* < 0.05.

Abbreviations: CI, confidence interval; H&Y, Hoehn and Yahr stage; MMSE, mini‐mental state examination; PD, Parkinson's disease; t‐α‐syn, total alpha‐synuclein; UPDRS III, Unified Parkinson's Disease Rating Scale Part III.

^a^ pS129‐α‐syn and t‐α‐syn were transformed as described in the “Methods” section.

### 3.2 Biomarker Comparisons Between Diagnostic Groups

**FIGURE 1:**
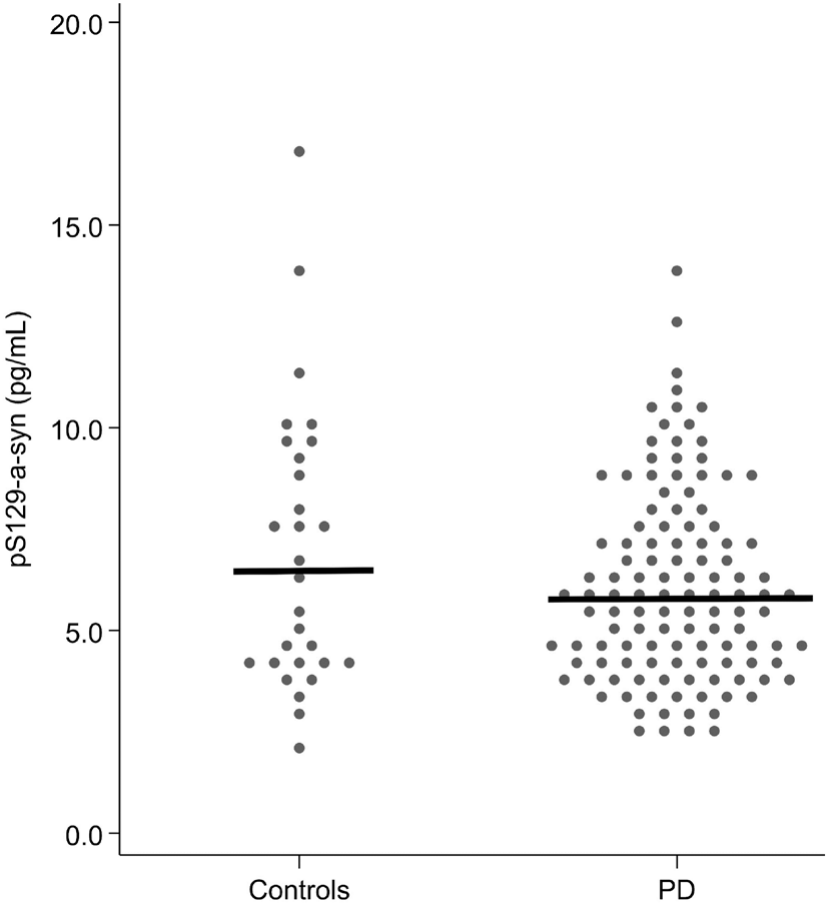
Boxplot showing the CSF concentration of pS129‐α‐syn in controls and patients with PD.

No significant differences in pS129‐α‐syn and the pS129‐α‐syn/t‐α‐syn ratio were observed between patients and controls in unadjusted analysis (Table 1, Figure 1). pS129‐α‐syn was significantly correlated with age (Figure S3). Using robust linear regression adjusted for age and sex, the results for both pS129‐α‐syn and pS129‐α‐syn/t‐α‐syn remained nonsignificant (Table 3). Similarly, after the exclusion of patients with CSF hemoglobin contamination, the results for pS129α‐syn remained nonsignificant (Table 3). Levels of t‐α‐syn differed between the groups, with lower levels in patients (*p* = 0.039; Table 1) as reported earlier [18]. This result remained significant after adjusting for age and sex (Table 3).

**TABLE 3:** Baseline comparison of CSF biomarker values in patients with PD compared to controls, using robust linear regression.

Biomarkers	All^a^			Hemoglobin < 200 ng/mL		
	*β* (95% CI)	*p*	*R* ^2^	β (95% CI)	*p*	*R* ^2^
pS129‐α‐syn^b^, ^c^	−0.09 (−0.26 to 0.07)	0.270	0.13	−0.10 (−0.27 to 0.07)	0.256	0.16
t‐α‐syn^b^, ^c^	—	—	—	−0.22 (−0.41 to −0.03)	**0.027**	0.10
pS129‐α‐syn/t‐α‐syn^c^	—	—	—	−0.01 (−0.23 to 0.20)	0.905	0.02

*Note:* Bold *p* values are statistically significant at *p* < 0.05.

Abbreviations: CI, confidence interval; pS129‐α‐syn, phosphorylated alpha‐synuclein at serine 129; t‐α‐syn, total alpha‐synuclein.

^a^ t‐α‐syn measurements were only available for samples with hemoglobin > 200 ng/mL.

^b^ pS129‐α‐syn and t‐α‐syn were transformed as described in the “Methods” section.

^c^ Analyses were adjusted for age and sex.

### 3.3 Association of Baseline CSF Biomarkers With Motor and Cognitive Impairment

Baseline pS129‐α‐syn and pS129‐α‐syn/t‐α‐syn were not associated with the annual change in UPDRS III or MMSE (Table 4). The results were unchanged when only including samples with hemoglobin levels below 200 ng/mL for pS129‐α‐syn (PD *N* = 85 and NC *N* = 29).

**TABLE 4:** Association of baseline CSF biomarkers with clinical assessment scores over 10 years in patients with PD.

Clinical assessments^a^,^b^	Main effect^c^ β (95% CI)	*p*	Interaction with time^c^ β (95% CI)	*p*	AIC	Marginal pseudo‐*R* ^2^	Conditional pseudo‐*R* ^2^
*pS129‐α‐syn*							
UPDRS III	−3.36 (−7.53 to 0.81)	0.114	0.81 (−0.10 to 1.73)	0.082	7314.6	0.18	0.71
Adjusted for LED	−2.98 (−7.31 to 1.36)	0.178	0.57 (−0.31 to 1.46)	0.202	7221.1	0.26	0.80
MMSE	1.95 (−4.49 to 8.40)	0.552	−0.45 (−1.86 to 0.97)	0.534	5245.5	0.20	0.62
*pS129‐α‐syn/t‐α‐syn*							
UPDRS III	0.91 (−0.97 to 2.78)	0.343	−0.15 (−0.65 to 0.34)	0.549	4721.8	0.16	0.75
Adjusted for LED	0.66 (−1.34 to 2.66)	0.518	−0.15 (−0.63 to 0.32)	0.529	4670.1	0.24	0.81
MMSE	1.03 (−2.02 to 4.08)	0.508	0.25 (−0.45 to 0.95)	0.489	3369.5	0.62	0.21

*Note:* Bold *p* values are statistically significant at *p* < 0.05. Models are adjusted for age at baseline and sex. The model including MMSE was also adjusted for years of education at baseline.

Abbreviations: AIC, akaike information criterion; CI, confidence interval; LED, total levodopa equivalent dose; MMSE, Mini‐mental state examination; pS129‐α‐syn, Phosphorylated alpha‐synuclein at serine 129; t‐α‐syn, total alpha‐synuclein; UPDRS III, Unified Parkinson's Disease Rating Scale Part III.

^a^ Transformations of MMSE, pS129‐α‐syn, and t‐α‐syn were applied, as described in the “Methods” section. The pS129‐α‐syn/t‐α‐syn was included in the models as a percentage.

^b^ Analyses for t‐α‐syn and the pS129‐α‐syn/t‐α‐syn ratio were limited to patients with hemoglobin levels below 200 ng/mL.

^c^ Main effect indicates the effect of pS129‐α‐syn levels on the intercept, and the interaction with time indicates the effect of pS129‐α‐syn levels on the slope (annual change per year) of the model.

### 3.4 pS129‐α‐Synuclein Assay

A detailed description of the assay development can be found in the Supporting Information. In brief, the developed assay is highly sensitive with an LLOQ well below the observed native levels in PD and control CSF samples, and specific for pS129‐α‐syn, as demonstrated by no reactivity with nonphosphorylated t‐α‐syn (Figure S1). The final assay utilizes a two‐fold assay sample dilution to minimize potential matrix effect while allowing clear separation from background in samples with lower levels of endogenous pS129‐α‐syn. Parallelism experiments showed a trend of decreasing recovery with increasing dilution factor, as concentrations approached the limit of quantification (Figure S2). Spike‐recovery experiments showed small overestimates of lower concentrations and underestimates of higher concentrations (Table S1).

## 4 Discussion

Over the past years, interest in pS129‐α‐syn as a biomarker for PD has increased, but readily available assays are scarce. Additionally, there is no consensus among previous studies. The relatively low abundance of pS129‐α‐syn in CSF requires highly sensitive detection methods. We therefore developed an assay on the ultrasensitive SIMOA platform for the quantification of pS129‐α‐syn in the CSF from 120 newly diagnosed PD patients of a longitudinal cohort and 29 controls.

### 4.1 pS129‐α‐Synuclein in CSF of PD Patients and Controls

Our in‐house assay exhibits a similar range of observed pS129‐α‐syn levels in CSF as the two other recently developed assays utilizing highly sensitive detection platforms [14, 17]. We found no significant differences in pS129‐α‐syn levels in patients with PD compared to controls, which is in line with a number of previous studies [10, 11, 12, 13, 14, 15]. However, three studies have reported higher [7, 8, 9] and one lower [17] levels in PD patients, with substantial overlap between groups. Of note, an assay first introduced in 2016 initially detected higher pS129‐α‐syn in PD patients [8], but no significant differences in later studies [11, 12, 15, 28]. Together, these findings do not support the clinical use of pS129‐α‐syn as a diagnostic biomarker for PD.

Because the majority of α‐syn in Lewy bodies is phosphorylated at serine 129, pS129‐α‐syn has been closely associated with PD pathology. However, we found no significant associations between pS129‐α‐syn levels and motor or cognitive impairment at the time of PD diagnosis. Furthermore, in longitudinal analyses, we found no association with motor or cognitive decline over time, despite up to 10 years of clinical follow‐up.

We observed positive correlations between age and pS129‐α‐syn levels both in the control and the PD patient groups. For NfL, age‐dependent cut‐off values have been proposed [29], a concept that might also be applicable to pS129‐α‐syn.

### 4.2 Other CSF Biomarkers in PD Patients and Controls

Using previously reported t‐α‐syn measurements from 75 patients in the same cohort [18], we evaluated the diagnostic potential of the pS129‐α‐syn/t‐α‐syn ratio but found no differences between patients and controls. This result contrasts with previous studies that have found that the ratio is significantly higher in patients with PD than in controls, despite finding no differences in pS129‐α‐syn alone [8, 11, 12]. We further examined the prognostic utility of this ratio; however, no significant associations were identified between pS129‐α‐syn/t‐α‐syn and motor or cognitive decline over time.

### 4.3 pS129‐α‐Synuclein Assay

The LLOQ of our assay is comparable to that of another recently published SIMOA assay for pS129‐α‐syn (0.6618 pg/mL) [17]. Both assays use a different antibody pair and antibody orientation. Our assay first captures pS129‐α‐syn and then detects this with a t‐α‐syn‐specific antibody, while the other SIMOA assay captures t‐α‐syn and then detects pS129‐α‐syn. During development, we also tested the reverse antibody pair orientation on the MSD platform, which yielded a higher LLOQ (data not shown).

### 4.4 Limitations and Strengths

This study has limitations. When testing the clinical utility of our assay, the small number of control samples may limit the power to detect between‐group differences with smaller effect sizes. On the technical side, parallelism and spike recovery marginally exceeded the recommended limits of 20%. Parallelism testing is ideally performed with samples with high endogenous analyte concentrations, which were not available. Therefore, only a few parallelism dilutions were possible before approaching the LLOQ. However, this does not change the ability of the in‐house assay to reliably quantify pS129‐α‐syn in CSF, since the main purpose of these tests is to evaluate differences in the matrix of calibrators and the matrix of the CSF samples. Apart from this, the assay shows high specificity, sensitivity, and precision, successfully quantifying pS129‐α‐syn in all included samples, with concentrations well above the limit of quantification. Lastly, to our knowledge, we are the first group to study the effect of CSF pS129‐α‐syn and the pS129‐α‐syn/t‐α‐syn ratio on the development of cognitive or motor decline over time. Therefore, our findings need to be confirmed in independent cohorts.

Our study also has notable strengths. First, the assay uses only commercially available components, which allows assay implementation in other laboratories. Next, we used standardized preanalytical procedures for obtaining and storing CSF samples. Another strength is that we included patients from the population‐based ParkWest cohort, who were diagnosed using established diagnostic criteria. In addition, the long follow‐up period with repeated assessments over time increases the accuracy of PD diagnosis. Population‐based studies reflect the real‐world population of PD more closely than clinic‐based studies and provide important information regarding age‐dependent outcomes, such as progression of disease [30, 31]. Lastly, because the patients included in our study were newly diagnosed, we were able to assess pS129‐α‐syn as a biomarker of both diagnosis and prognosis in the earliest clinical stages of PD.

## 5 Outlook

We present an ultrasensitive in‐house assay that can reliably quantify pS129‐α‐syn in CSF from patients with PD and controls. Our study adds to the body of evidence for the limited use of pS129‐α‐syn as a diagnostic and prognostic biomarker in research and clinical practice.

## Author Contributions

**Camilla Christina Pedersen:** conceptualization, formal analysis, funding acquisition, investigation (data from the validation process), methodology (SIMOA assay), writing – original draft preparation. **Guido Alves** and **Ole‐Bjørn Tysnes:** data curation (clinical/demographic), investigation, writing – review and editing. **Jodi Maple‐Grødem** and **Johannes Lange:** conceptualization, funding acquisition, supervision, writing – review and editing. **Johannes Lange:** data curation (biological), Investigation (quantification of pS129‐α‐syn in patient and control samples). All authors read and approved the final manuscript and agreed to be accountable for their contributions.

## Conflicts of Interest

C.C.P. is supported by the Western Norway Regional Health Authority (29604) and the Norwegian Parkinson Research Foundation. O.B.T. reports no disclosures relevant to the manuscript. G.A. and J.M.G. are supported by the Research Council of Norway (287842). J.M.G. and J.L. are supported by the Nasjonalforeningen for Folkehelsen (14846 and 16152).

## Supporting information


**Figure S1.** Calibration curves for pS129‐α‐syn and t‐α‐syn. Recombinant protein standards were diluted in a four‐fold series. A 4PL curve fit with 1/y^2^ weighting was applied to each curve. AEB: Average enzyme per bead; pS129‐α‐syn: phosphorylated alpha‐synuclein at serine 129; t‐α‐syn: total alpha‐synuclein.
**Figure S2**. Parallelism of CSF samples prepared as a two‐fold serial dilution.
**Table S1**. Spike recovery of four CSF samples.
**Table S2**. Intra‐ and interassay CVs of four CSF samples.
**Figure S3**. Scatterplots of age at baseline against baseline CSF pS129‐α‐syn in (A) controls (*R* ^2^ = 0.16, *p* = 0.047) and (B) patients with PD (*R* ^2^ = 0.12, *p* < 0.001). *R* ^2^ and *p* values are based on robust linear regression and calculated as described in the Methods section.

## Data Availability

The data that support the findings of this study are available on request from the corresponding author. The data are not publicly available due to privacy or ethical restrictions.

## References

[1] P. Calabresi , G. Di Lazzaro , G. Marino , F. Campanelli , and V. Ghiglieri , “Advances in Understanding the Function of Alpha‐Synuclein: Implications for Parkinson's Disease,” Brain 146, no. 9 (2023): 3587–3597, 10.1093/brain/awad150. 37183455 PMC10473562

[2] M. G. Spillantini , M. L. Schmidt , V. M. Lee , J. Q. Trojanowski , R. Jakes , and M. Goedert , “Alpha‐Synuclein in Lewy Bodies,” Nature 388, no. 6645 (1997): 839–840, 10.1038/42166. 9278044

[3] D. J. Gelb , E. Oliver , and S. Gilman , “Diagnostic Criteria for Parkinson Disease,” Archives of Neurology 56, no. 1 (1999): 33–39, 10.1001/archneur.56.1.33. 9923759

[4] J. P. Anderson , D. E. Walker , J. M. Goldstein , et al., “Phosphorylation of Ser‐129 Is the Dominant Pathological Modification of α‐Synuclein in Familial and Sporadic Lewy Body Disease*,” Journal of Biological Chemistry 281, no. 40 (2006): 29739–29752, 10.1074/jbc.M600933200. 16847063

[5] H. Fujiwara , M. Hasegawa , N. Dohmae , et al., “α‐Synuclein Is Phosphorylated in Synucleinopathy Lesions,” Nature Cell Biology 4, no. 2 (2002): 160–164, 10.1038/ncb748. 11813001

[6] C. C. Pedersen , J. Maple‐Grødem , and J. Lange , “A Systematic Review of Biofluid Phosphorylated α‐Synuclein in Parkinson's Disease,” Parkinsonism & Related Disorders 132 (2024): 107240, 10.1016/j.parkreldis.2024.107240. 39721932

[7] L. P. Oosterveld , I. M. W. Verberk , N. K. Majbour , et al., “CSF or Serum Neurofilament Light Added to α‐Synuclein Panel Discriminates Parkinson's From Controls,” Movement Disorders 35, no. 2 (2019): 288–295, 10.1002/mds.27897. 31737952 PMC7027879

[8] N. K. Majbour , N. N. Vaikath , K. D. van Dijk , et al., “Oligomeric and Phosphorylated Alpha‐Synuclein as Potential CSF Biomarkers for Parkinson's Disease,” Molecular Neurodegeneration 11 (2016): 1–15, 10.1186/s13024-016-0072-9. 26782965 PMC4717559

[9] Y. Wang , M. Shi , K. A. Chung , et al., “Phosphorylated α‐Synuclein in Parkinson's Disease,” Science Translational Medicine 4, no. 121 (2012): 3002566, 10.1126/scitranslmed.3002566. PMC3302662 22344688

[10] I. Schulz , N. Kruse , R. G. Gera , et al., “Systematic Assessment of 10 Biomarker Candidates Focusing on α‐Synuclein‐Related Disorders,” Movement Disorders 36, no. 12 (2021): 2874–2887, 10.1002/mds.28738. 34363416

[11] N. K. Majbour , J. O. Aasly , E. Hustad , et al., “CSF Total and Oligomeric α‐Synuclein Along With TNF‐α as Risk Biomarkers for Parkinson's Disease: A Study in LRRK2 Mutation Carriers,” Translational Neurodegeneration 9, no. 1 (2020): 15, 10.1186/s40035-020-00192-4. 32375873 PMC7201744

[12] I. van Steenoven , N. K. Majbour , N. N. Vaikath , et al., “α‐Synuclein Species as Potential Cerebrospinal Fluid Biomarkers for Dementia With Lewy Bodies,” Movement Disorders 33, no. 11 (2018): 1724–1733, 10.1002/mds.111. 30440090 PMC6519232

[13] P. G. Foulds , O. Yokota , A. Thurston , et al., “Post Mortem Cerebrospinal Fluid α‐Synuclein Levels Are Raised in Multiple System Atrophy and Distinguish This From the Other α‐Synucleinopathies, Parkinson's Disease and Dementia With Lewy Bodies,” Neurobiology of Disease 45, no. 1 (2012): 188–195, 10.1016/j.nbd.2011.08.003. 21856424 PMC3657198

[14] A. M. Silva , E. S. Hickford , and P. Cutler , “An Immunoassay for the Quantification of Phosphorylated α‐Synuclein at Serine 129 in Human Cerebrospinal Fluid,” Bioanalysis 16 (2024): 1125–1139, 10.1080/17576180.2024.2407718. 39404180 PMC11583607

[15] N. K. Majbour , I. Y. Abdi , M. Dakna , et al., “Cerebrospinal α‐Synuclein Oligomers Reflect Disease Motor Severity in DeNoPa Longitudinal Cohort,” Movement Disorders 36, no. 9 (2021): 2048–2056, 10.1002/mds.28611. 33978256

[16] D. M. Rissin , C. W. Kan , T. G. Campbell , et al., “Single‐Molecule Enzyme‐Linked Immunosorbent Assay Detects Serum Proteins at Subfemtomolar Concentrations,” Nature Biotechnology 28, no. 6 (2010): 595–599, 10.1038/nbt.1641. PMC2919230 20495550

[17] M. Norman , T. Gilboa , and D. R. Walt , “High‐Sensitivity Single Molecule Array Assays for Pathological Isoforms in Parkinson's Disease,” Clinical Chemistry 68, no. 3 (2022): 431–440, 10.1093/clinchem/hvab251. 35064661

[18] M. G. Førland , O. B. Tysnes , D. Aarsland , et al., “The Value of Cerebrospinal Fluid α‐Synuclein and the Tau/α‐Synuclein Ratio for Diagnosis of Neurodegenerative Disorders With Lewy Pathology,” European Journal of Neurology 27, no. 1 (2020): 43–50, 10.1111/ene.14032. 31293044

[19] G. Alves , B. Muller , K. Herlofson , et al., “Incidence of Parkinson's Disease in Norway: The Norwegian ParkWest Study,” Journal of Neurology, Neurosurgery, and Psychiatry 80, no. 8 (2009): 851–857, 10.1136/jnnp.2008.168211. 19246476

[20] A. J. Hughes , S. E. Daniel , L. Kilford , and A. J. Lees , “Accuracy of Clinical Diagnosis of Idiopathic Parkinson's Disease: A Clinico‐Pathological Study of 100 Cases,” Journal of Neurology, Neurosurgery, and Psychiatry 55, no. 3 (1992): 181–184, 10.1136/jnnp.55.3.181. 1564476 PMC1014720

[21] S. Fahn , “Unified Parkinson's Disease Rating Scale,” Recent Developments in Parkinsons Disease 2 (1987): 153–163.

[22] M. M. Hoehn and M. D. Yahr , “Parkinsonism: Onset, Progression and Mortality,” Neurology 17, no. 5 (1967): 427–442, 10.1212/wnl.17.5.427. 6067254

[23] M. F. Folstein , S. E. Folstein , and P. R. McHugh , “Mini‐Mental State. A Practical Method for Grading the Cognitive State of Patients for the Clinician,” Journal of Psychiatric Research 12, no. 3 (1975): 189–198, 10.1016/0022-3956(75)90026-6. 1202204

[24] G. Alves , K. Brønnick , D. Aarsland , et al., “CSF Amyloid‐Beta and Tau Proteins, and Cognitive Performance, in Early and Untreated Parkinson's Disease: The Norwegian ParkWest Study,” Journal of Neurology, Neurosurgery, and Psychiatry 81, no. 10 (2010): 1080–1086, 10.1136/jnnp.2009.199950. 20547614

[25] M. G. Førland , A. Öhrfelt , L. S. Oftedal , et al., “Validation of a New Assay for α‐Synuclein Detection in Cerebrospinal Fluid,” Clinical Chemistry and Laboratory Medicine 55, no. 2 (2016): 254–260, 10.1515/cclm-2016-0409. 27474841

[26] V. Philipps , H. Amieva , S. Andrieu , et al., “Normalized Mini‐Mental State Examination for Assessing Cognitive Change in Population‐Based Brain Aging Studies,” Neuroepidemiology 43, no. 1 (2014): 15–25, 10.1159/000365637. 25248074

[27] “R2_NAKAGAWA: Stata Module for Computing Nakagawa's R‐Squared Statistic for Multilevel Mixed‐Effects Linear Regression. Version S459345.” Boston College Department of Economics. https://ideas.repec.org/c/boc/bocode/s459345.html 2024.

[28] V. C. Constantinides , N. K. Majbour , G. P. Paraskevas , et al., “Cerebrospinal Fluid α‐Synuclein Species in Cognitive and Movements Disorders,” Brain Sciences 11, no. 1 (2021): 119, 10.3390/brainsci11010119. 33477387 PMC7830324

[29] L. Vermunt , M. Otte , I. M. W. Verberk , et al., “Age‐ and Disease‐Specific Reference Values for Neurofilament Light Presented in an Online Interactive Support Interface,” Annals of Clinical Translational Neurology 9, no. 11 (2022): 1832–1837, 10.1002/acn3.51676. 36196979 PMC9639622

[30] A. D. Macleod , R. Henery , P. C. Nwajiugo , N. W. Scott , R. Caslake , and C. E. Counsell , “Age‐Related Selection Bias in Parkinson's Disease Research: Are We Recruiting the Right Participants?,” Parkinsonism & Related Disorders 55 (2018): 128–133, 10.1016/j.parkreldis.2018.05.027. 29871791

[31] B. K. Beaulieu‐Jones , F. Frau , S. Bozzi , et al., “Disease Progression Strikingly Differs in Research and Real‐World Parkinson's Populations,” MedRxiv 10 (2024): 58, 10.1101/2024.02.17.24302981. PMC10937726 38480700

