# Supplementary material for: Cerebrospinal Fluid Phosphorylated Alpha‐Synuclein in Newly Diagnosed Parkinson's Disease
Source: Eur J Neurol. 2025 Jun 2;32(6):e70167. doi: 10.1111/ene.70167 (PMC12128147; doi:10.1111/ene.70167)
Supplement: Supplementary file 1 — Figure S1. Calibration curves for pS129‐α‐syn and t‐α‐syn. Recombinant protein standards were diluted in a four‐fold series. A 4PL curve fit with 1/y2 weighting was applied to each curve. AEB: Average enzyme per bead; pS129‐α‐syn: phosphorylated alpha‐synuclein at serine 129; t‐α‐syn: total alpha‐synuclein. Figure S2. Parallelism of CSF samples prepared as a two‐fold serial dilution. Table S1. Spike recovery of four CSF samples. Table S2. Intra‐ and interassay CVs of four CSF samples. Figure S3. Scatterplots of age at baseline against baseline CSF pS129‐α‐syn in (A) controls (R 2 = 0.16, p = 0.047) and (B) patients with PD (R 2 = 0.12, p < 0.001). R 2 and p values are based on robust linear regression and calculated as described in the Methods section. [file ENE-32-e70167-s001.docx]

**Supporting Methods and Results**

**Assay development**

*Reagent preparations*

Bead coupling and biotinylation were performed according to the manufacturer’s instructions (Quanterix, Billerica, MA, USA), with minor alterations.

The capture antibody was buffer exchanged to bead conjugation buffer (#101357; Quanterix, using an Amicon Ultra-0.5 mL (50 kDa) centrifugal filter (#UFC505024; Merck Millipore Ltd., Carrigtwohill, County Cork, Ireland) with six repetitions. The capture antibody was available in a carrier-free formulation and did not require a purification step like BSA-removal prior to coupling to the beads. Next, 1.4 x 10^9^ beads/mL of 2.7 µm Alexa™488-coupled carboxylated paramagnetic beads (#103526; Quanterix) were washed with bead wash buffer (#101355; Quanterix,), before being resuspended in bead conjugation buffer. The beads were then incubated with 0.3 mg/mL 1-ethyl-3-(3-dimethylaminopropyl) carbodiimide (EDC; ThermoFisher Scientific, Rockford, IL, USA) for 30 min at 4°C, with rotation and shaking. After washing, the beads were incubated with the buffer exchanged capture antibody for 2 h at 4°C, with rotation and shaking. The beads were incubated with bead blocking buffer (#101356; Quanterix) for 45 min at 4°C. The beads were stored in the dark at 4°C in bead diluent (#101362; Quanterix) until use.

The final detector antibody was available in carrier-free formulation in PBS and did not require preparation. Other antibodies were buffer exchanged to PBS pH 7 using an Amicon Ultra-0.5 mL (50 kDa) centrifugal filter as described above. A 1 mg vial of EZ-Link NHS-PEG4-Biotin (#A39259; ThermoFisher Scientific) was resuspended in 100 µL distilled water and quickly added to the detector antibody in PBS at a molar excess of 40x. The biotin and detector antibody (biotinylation reaction) were incubated for 30 min at room temperature. To remove excess biotin, the reaction was buffer exchanged to PBS using an Amicon Ultra-0.5 mL (50 kDa) centrifugal filter and six repetitions. The final antibody concentration was determined using the Qubit 2.0 Fluorometer, and the antibody was stored at 4°C.

We optimized the protocol recommended by the manufacturer. The final assay uses the 3-step protocol, Alexa™488-coupled paramagnetic beads (#103526; Quanterix), and Sample Diluent A (#101575; Quanterix) as assay buffer.

CSF samples for method development

The CSF samples were excess aliquots from clinical routine lumbar puncture. De-identification of all samples was completed according to the Norwegian biobanking laws. Samples were centrifuged at 2000 xg for 10 min prior to being stored in polypropylene tubes at -80°C. Later, samples were subjected to a single freeze-thaw event before their analysis, where they were divided into two sets of aliquots: 70 µL aliquots for precision evaluation, and 220 µL aliquots for the remaining validation steps. All aliquots were stored at -80°C.

Antibodies

Combinations of antibodies were initially screened using an electrochemiluminescence ELISA (MSD Discovery, USA), using a similar approach as described previously.^1^ This allows for faster screening using less material. In brief, a total of 14 antibodies specific for total α-syn or pS129-α-syn were assessed for their performance as capture and/or detector antibody measuring recombinant pS129-α-syn (Proteos; Kalamazoo, MI, USA) at concentrations between 160,000 and 2.4 pg/mL. Selection criteria were low background signal, and the highest possible, increasing signal-to-noise ratio for the different calibrator concentrations. Four promising antibody combinations were transferred to the SIMOA platform for further testing, with the capture antibodies coupled to paramagnetic beads and detector antibodies undergoing biotinylation. The best antibody pair that we identified was anti-phospho-α-synuclein Rabbit monoclonal antibody (#87281; Cell Signaling Technology, Danvers, MA, USA) as the capture antibody and anti-alpha-synuclein Mouse monoclonal antibody (#Ab280382; Abcam, Cambridge, UK) as the detector antibody. We proceeded to optimize this combination on the SR-X platform and validated the assay based on the characteristics below.

## Validation

We performed a set of assay validation experiments based on published guidelines (Andreasson et al.).^2^

Specificity for pS129-α-syn

We assessed the specificity for pS129-α-syn assay by measuring a 4-fold serial dilution of non-phosphorylated recombinant α-syn (#S-1001-1; rPeptide, Bogart, GA, USA) in Sample Diluent A, ranging from 0.6 to 2 500 pg/mL.

Our assay showed no cross-reactivity with t-α-syn (**Supplementary Figure 1**), which demonstrates specificity to pS129-α-syn.


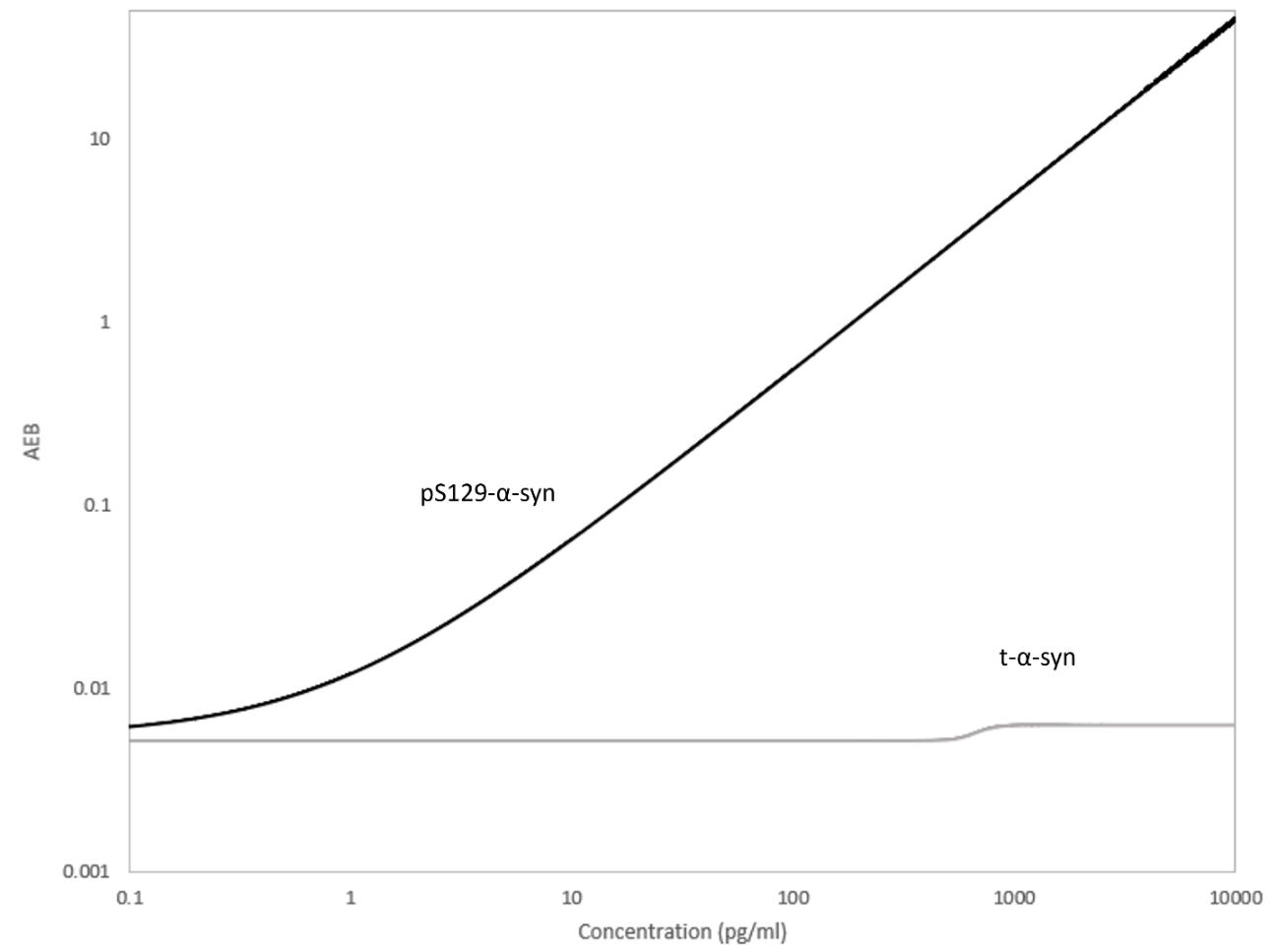


**Supplementary Figure 1.** Calibration curves for pS129-α-syn and t-α-syn. Recombinant protein standards were diluted in a four-fold series. A 4PL curve fit with 1/y^2^ weighting was applied to each curve. AEB: Average enzyme per bead; pS129-α-syn: phosphorylated alpha-synuclein at serine 129; t-α-syn: total alpha-synuclein.

Parallelism

The purpose of this test is to assess the alignment of the dose-response curve in test samples and the calibrator samples in the range of endogenous levels. A dilution series of CSF samples is produced, measured, and the recovery-% relative to a reference dilution calculated. Four CSF samples with endogenous pS129-α-syn levels between 8.3 and 17.2 pg/ml were diluted in a 2-fold series in Sample Diluent A down to 16-fold. All undiluted samples and their dilutions were analyzed in duplicate.

The final assay sample dilution was 2-fold and all pS129-α-syn concentrations were normalized to this dilution (**Supplementary Figure 2**). Ideally, recovery range is between 80% and 120%. We observed a trend to reduced recovery with increasing dilution and higher variance as levels came closer to the limit of detection. The method development samples had pS129-α-syn levels comparable to typical PD and normal control samples.


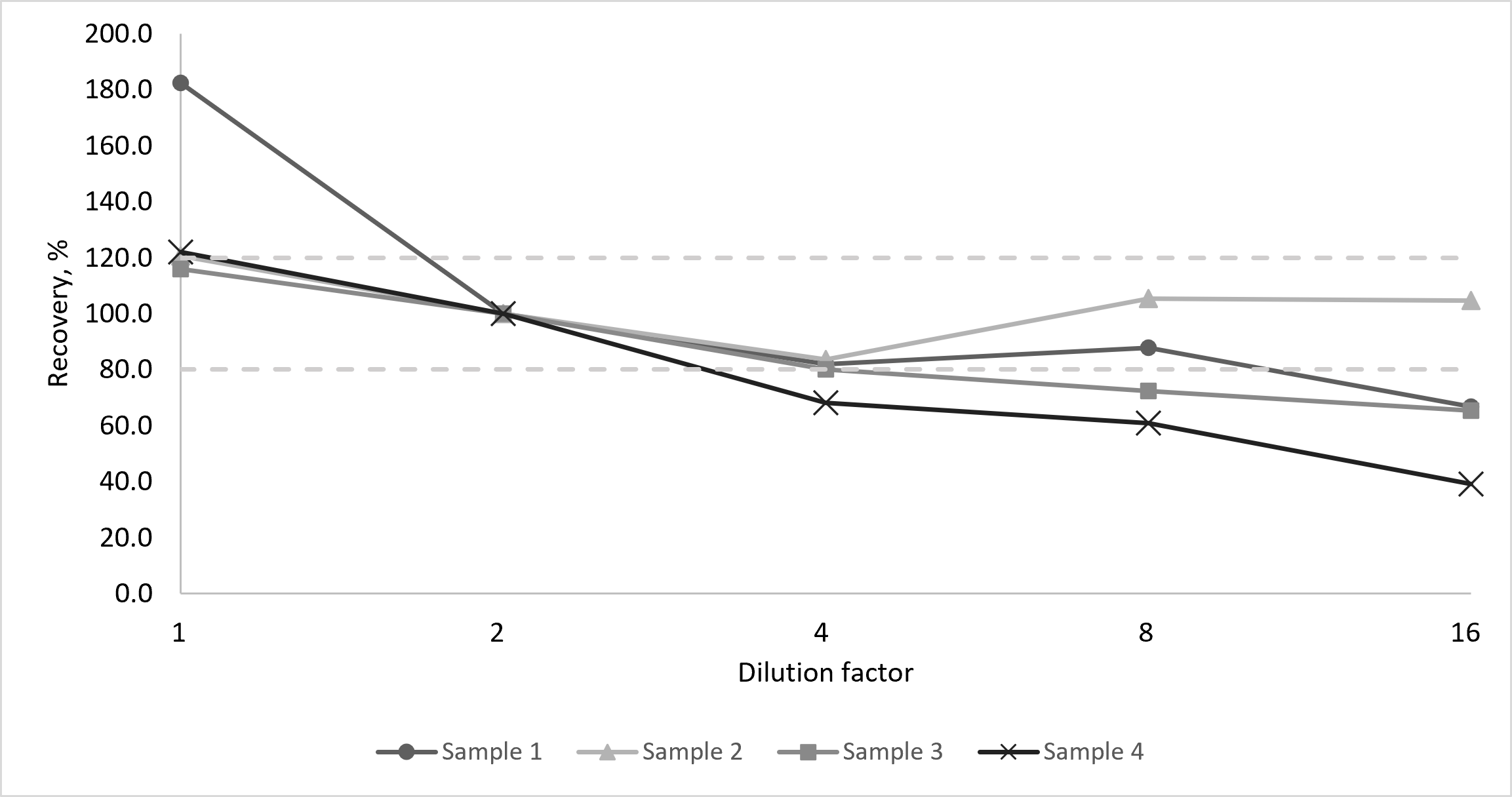


**Supplementary Figure 2.** Parallelism of CSF samples prepared as a two-fold serial dilution.

Recovery for undiluted (neat) samples dropped compared to standard assay dilutions, suggesting a potential matrix effect in undiluted samples.

Spike-recovery

Four samples of 2-fold diluted CSF were spiked with pS129-α-syn calibrator to a final concentration of 9.8, 39.1, and 156.3 pg/mL by adding 1 volume of pS129-α-syn calibrator and 1 volume Sample Diluent A to 2 volumes of CSF. The concentrations of pS129-α-syn from the diluted calibrator were determined by subtracting the concentrations of the unspiked CSF from the spiked CSF. Calculation of the percent recovery was then performed.

Three different concentrations of calibrator were spiked into four CSF samples. The recovery rate of the spiking calibrators was between 84.2% and 126.3%. The CV values for the recovery of all spiking concentrations were between 5.6 and 15.1% (**Supplementary Table 1**). While the lower spikes gained a recovery above 110%, the higher spike had a recovery around 85%.

**Supplementary Table 1**. Spike-recovery of four CSF samples.

| **Name** | **Spike, pg/mL** | | |
| --- | --- | --- | --- |
|  | **9.8** | **39.1** | **156.3** |
|  | **Recovery, %** | | |
| CSF 1 | 128.8 | 130.1 | 88.5 |
| CSF 2 | 96.4 | 118.0 | 77.7 |
| CSF 3 | 121.5 | 140.4 | 86.6 |
| CSF 4 | 96.7 | 116.7 | 84.0 |
|  |  |  |  |
| **Mean** | **110.8** | **126.3** | **84.2** |
| SD | 16.8 | 11.2 | 4.7 |
| CV, % | 15.1 | 8.8 | 5.6 |

Abbreviations. SD: Standard deviation; CV: Coefficient of variance.

This may indicate a slight difference in binding characteristics between the artificial pS129-α-syn peptide and endogenous pS129-α-syn. However, parallelism experiments based on raw AEB values and fitted concentrations yielded similar results (data not shown), indicating that these small discrepancies are not hampering the accuracy of the assay.

Precision

Four CSF samples were used to assess the precision of the assay. On five separate occasions, five aliquots of each sample were analyzed. A total of 25 aliquots were quantified for each sample. Precision replicates were individually diluted on the same day as the assay was performed.

The four CSF samples tested repetitively had intra-assay CVs between 11.9% and 21.3% and inter-assay CVs between 18.6% and 28.9% (Supplementary Table 2).

**Supplementary Table 2**. Intra-assay and inter-assay CVs of four CSF samples.

| **Name** | **Mean conc., pg/mL** | **Intra-assay CV, %** | **Inter-assay CV, %** |
| --- | --- | --- | --- |
| CSF 1 | 13.0 | 12.5 | 18.6 |
| CSF 2 | 6.9 | 11.9 | 18.9 |
| CSF 3 | 10.7 | 17.7 | 22.2 |
| CSF 4 | 16.2 | 21.3 | 28.9 |
|  |  |  |  |
| Mean | - | 15.9 | 22.2 |

Abbreviations. Conc: Concentration; CV: Coefficient of variance

Lower limit of quantification (LLOQ)

LLOQ was determined using coefficient of variance (CV) profiling (20% threshold) using the assay developer tool provided by Quanterix. The LLOQs from five separate occasions were averaged to obtain an overall LLOQ of 0.77 ± 0.17 (CV 21.8%). The LLOQ of the assay used to measure patient samples had a LLOQ of 0.57pg/ml.

## Statistical analysis

Assay data analysis

Standards were analyzed in triplicate and samples were measured in duplicate. A standard curve fit was applied to the calibration curves (4PL with 1/y^2^ weighting) and concentration calculations were performed using the SR-X instrument software version 1.2.0 (Quanterix). Signals for all standards and samples were measured in average enzyme per bead (AEB) units. Plotting of the calibration curves was performed using the 4PL Assay Developer Tool, provided by the manufacturer (Quanterix). Excel (Microsoft, Redmond, WA, USA) was used to perform all other calculations. For parallelism, the values of the concentrations were normalized to the values of the optimal assay dilution for easier comparison. The Excel sheet provided by Andreasson as a supplementary file was used to calculate intra- and inter-assay CVs for the precision samples, according to the ISO 5725-2 section 7.4.^2^ LLOQ was determined using the available 4PL Assay Developer Tool, provided by the manufacturer (Quanterix).


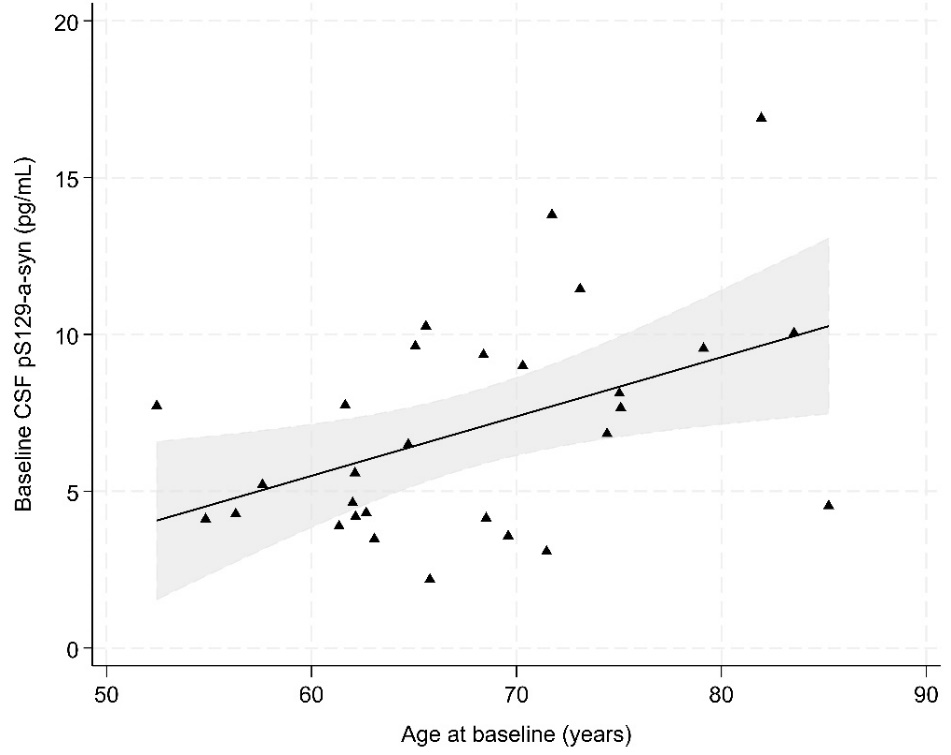
A)

B)
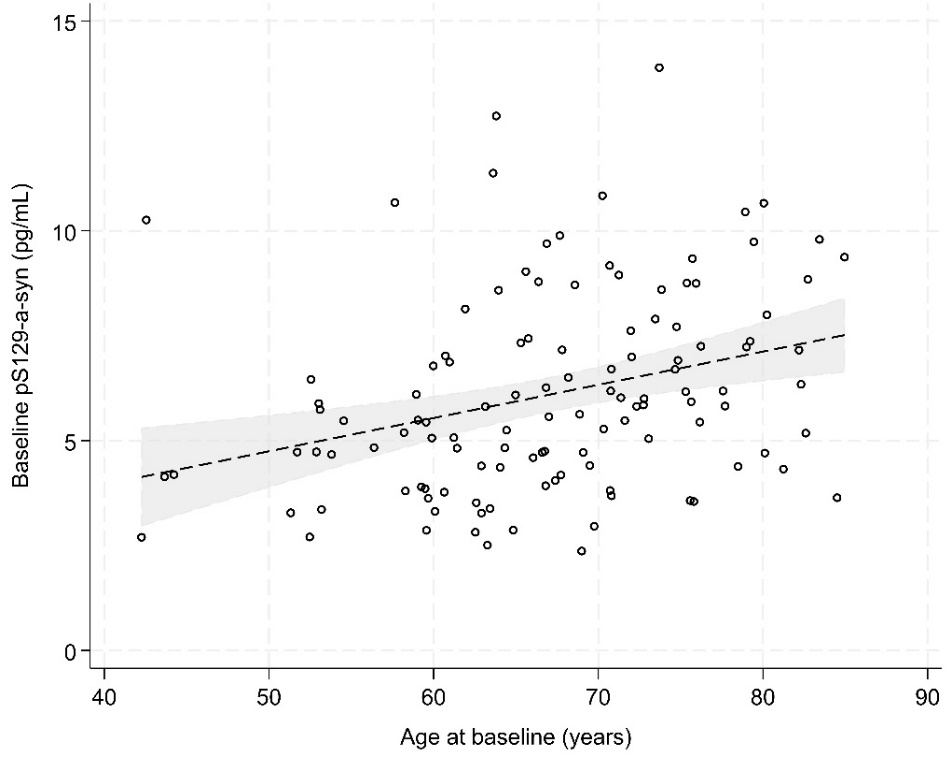


**Supplementary Figure 3.** Scatterplots of age at baseline against baseline CSF pS129-α-syn in A) controls (R^2^ = 0.16, p = 0.047) and B) patients with PD (R^2^ = 0.12, p < 0.001). R^2^ and p-values are based on robust linear regression and calculated as described in the Methods section.

# References

1 Førland, M. G. *et al.* Validation of a new assay for α-synuclein detection in cerebrospinal fluid. *Clin Chem Lab Med* **55**, 254-260 (2016). [https://doi.org:10.1515/cclm-2016-0409](rewritten://69b33c27-0de3-4df0-be53-993ff9dcb9ca)

2 Andreasson, U. *et al.* A Practical Guide to Immunoassay Method Validation. *Front Neurol* **6**, 179 (2015). [https://doi.org:10.3389/fneur.2015.00179](rewritten://17f12558-748d-4cec-9b98-c9d085b5ba09)
